# Fluorescence-Activated Cell Sorting Using the D-Root Device and Optimization for Scarce and/or Non-Accessible Root Cell Populations

**DOI:** 10.3390/plants9040499

**Published:** 2020-04-14

**Authors:** Mary-Paz González-García, Estéfano Bustillo-Avendaño, Alvaro Sanchez-Corrionero, Juan C. del Pozo, Miguel A. Moreno-Risueno

**Affiliations:** Centro de Biotecnología y Genómica de Plantas (Universidad Politécnica de Madrid—Instituto Nacional de Investigación y Tecnología Agraria y Alimentaria), Campus de Montegancedo, Pozuelo de Alarcón, 28223 Madrid, Spain; estefano.bustillo@upm.es (E.B.-A.); alvaro.scorrionero@upm.es (A.S.-C.); pozo@inia.es (J.C.d.P.); miguelangel.moreno@upm.es (M.A.M.-R.)

**Keywords:** FACS, root protoplasts, D-Root, cell-type, founder cells, lateral roots

## Abstract

Fluorescence-activated cell sorting (FACS) is a technique used to isolate specific cell populations based on characteristics detected by flow cytometry. FACS has been broadly used in transcriptomic analyses of individual cell types during development or under different environmental conditions. Different protoplast extraction protocols are available for plant roots; however, they were designed for accessible cell populations, which normally were grown in the presence of light, a non-natural and stressful environment for roots. Here, we report a protocol using FACS to isolate root protoplasts from Arabidopsis green fluorescent protein (GFP)-marked lines using the minimum number of enzymes necessary for an optimal yield, and with the root system grown in darkness in the D-Root device. This device mimics natural conditions as the shoot grows in the presence of light while the roots grow in darkness. In addition, we optimized this protocol for specific patterns of scarce cell types inside more differentiated tissues using the mCherry fluorescent protein. We provide detailed experimental protocols for effective protoplasting, subsequent purification through FACS, and RNA extraction. Using this RNA, we generated cDNA and sequencing libraries, proving that our methods can be used for genome-wide transcriptomic analyses of any cell-type from roots grown in darkness.

## 1. Introduction

Plants, as sessile organisms, are able to adapt to the environmental conditions due to their great plasticity to respond to a wide spectrum of external signals. The root system maintains vital functions such as the incorporation of water and nutrients, anchorage to the soil, and interaction with symbiotic organisms among others. Over the last few years, the capacity of the root system to respond to varying conditions in the rhizosphere has been shown to be critical for nutrition and response to adverse environmental conditions [1]. Therefore, changes in the root system can have an impact on nutrient assimilation capacity and, ultimately, on crop productivity [2].

The Arabidopsis root is a good model for the study of many processes and physiological responses that require the integration of different signaling pathways [3,4,5]. Roots are plant organs formed by different cell types that act coordinately to develop and respond specifically to environmental cues. Thus, external signals trigger molecular events that might be specific to each cell type to modulate their cell division/differentiation or physiological and hormonal responses. As a result, this cell-type-specialized root system is thought to respond more efficiently to different environmental changes. In nature, roots grow mainly below the soil in darkness; however, the majority of the transcriptomic analyses of cell types during development or under different environmental conditions have been performed with the roots grown in the presence of light [6,7,8]. Root illumination generates a stress that affects growth, hormonal signaling, abiotic responses, or nutrient starvation adaptation compared with roots grown in the dark [9,10]. Particularly, light signaling in roots affects auxin biosynthesis and transport, as this hormone has been proposed as a potential integrator of light signaling and root development [9,11,12]. In addition, light directly regulates root growth and modifies responses to other environmental signals [9,13,14].

New growing methods have been proposed to cultivate the roots in darkness, such as the use of dark agar plugs [15], black-colored vertical plates [16], and the D-Root system [17]. Previous results show that the use of the D-Root device is a reliable system to study root growth in response to different treatments or adverse environmental conditions without the influence of light in roots. Dark-grown root plants using the D-Root system showed longer main-roots and a higher number of lateral roots, while root hair length decreased [9]. The D-Root system consists of a black methacrylate box in which transparent in vitro plates are inserted. The light coming from the top is blocked by inserting a comb between the shoot and the root [17]. The D-Root system allows for the cultivation of the aerial part under a determined photoperiod while the root system is in darkness [17]. Another advantage of the D-Root system is that it enables the cultivation of a large number of seedlings in a simple and accurate way as compared to other available devices. As protoplasting and cell sorting studies require a large number of seedlings and material, the use of the D-Root device would mimic a more natural environment without compromising yield. Overall, the use of the D-Root device could identify novel mechanisms that may have been previously been overlooked by the influence of light. Eventually, this approach might be used to establish functional implications in crop models or transferred to other agronomical important species, as the D-Root device is scalable.

A critical step for analyzing cell types using transcriptomics is the efficient isolation of the cell types of interest. One of the most common methods used for specific cell-type isolation is fluorescent-activated cell sorting (FACS) [7,18,19,20,21,22]. FACS is a type of flow cytometry that allows the separation of individual cells based on their physical characteristics and/or their fluorescence. FACS is based on characteristics detected by a flow cytometer, which is coupled to a cell separator. Flow cytometry is a powerful technique used to distinguish and characterize different cell types, which allows researchers to rapidly, accurately, and simply collect data related to many parameters from a heterogeneous fluid mixture containing living cells [23,24,25]. This technique, which has been used primarily in biomedical and immunological research, uses lasers to discriminate different cell types in real time based on specific properties of the cells.

The use of FACS in plants involves the generation of protoplasts [26]. To efficiently filter protoplast through FACS, high-quality protoplast extraction, high yield, and integrity are required. Different methods have been used to determine protoplast viability using fluorescent dyes such as DAPI (4,6-diamidino-2-phenylindole) and PI (propidium iodide) for membrane integrity or cFDA (carboxifluorescein diaceate) to mark viable cells [27,28]. The main limitation in protoplast extraction is the use of a correct enzyme-mix combination to efficiently digest the plant cell wall without affecting cell integrity. Different enzymes have been reported as necessary for good protoplasting such as cellulase, pectolyase, and macerozyme. However, comparative studies explaining the requirements for these enzymes in combination with FACS are missing. Most protocols for FACS have been developed for accessible cell types, mostly those in the root meristem area, although cell types from more differentiated root zones have also been profiled [7,29,30]. Even if these cell types contain a low number of cells, for instance the quiescent center, a sufficient number of protoplasts can be obtained by increasing the number of seedlings processed [31]. Extraction of cell types in the most inner cell layers of the roots, such as pericycle cells (using the J0121 line) [32] can also be achieved by increasing seedling number [7]. In the fully mature zone of the root, cells have additional barriers due to changes in the cell wall composition, such as Casparian strip formation and cell wall modifications reported for the pericycle [33,34]. Casparian strips generate highly hydrophobic barriers that are more resistant to chemical and enzymatic degradation. Detailed protocols for extracting scarce cell types located within more differentiated or fully differentiated tissues, such as lateral root founder cells and lateral root cell types have not been established.

The main motivation of this work was to implement a protoplasting and FACS protocol using the D-Root device to allow for the performance of transcriptomics analyses of cell types in an environment similar to nature. In addition, these protocols were optimized to use the minimum number/amount of enzymes, but also to extract scarce and/or non-accessible cell type populations, which are not easily accessible because of their location inside mature root tissues. 

## 2. Results

To optimize the protoplasting protocol followed by FACS, we cultivated 12 × 12 cm plates containing *Arbidopsis* seedlings under standard conditions (long day photoperiod, 22 °C) for 6 days using the D-Root system. Please, check the video in [17] to find out how the D-Root system is assembled. Based on our experience while implementing this protocol, we determined that by using five D-root plates, a sufficient number of positive protoplasts (~2000) could be obtained for all tested GFP marker lines. Particularly, we tested the lines *pWER:ER-GFP*, J3411, J0571, *pSHR:ER-GFP,* and J0661, which marked epidermis, lateral root cap, ground tissue, the stele, and the whole pericycle of the root apical meristem, respectively. Here, we report results for the *pWER:ER-GFP* line but we obtained similar results for all these mentioned GFP-marked lines.

**Figure 1:**
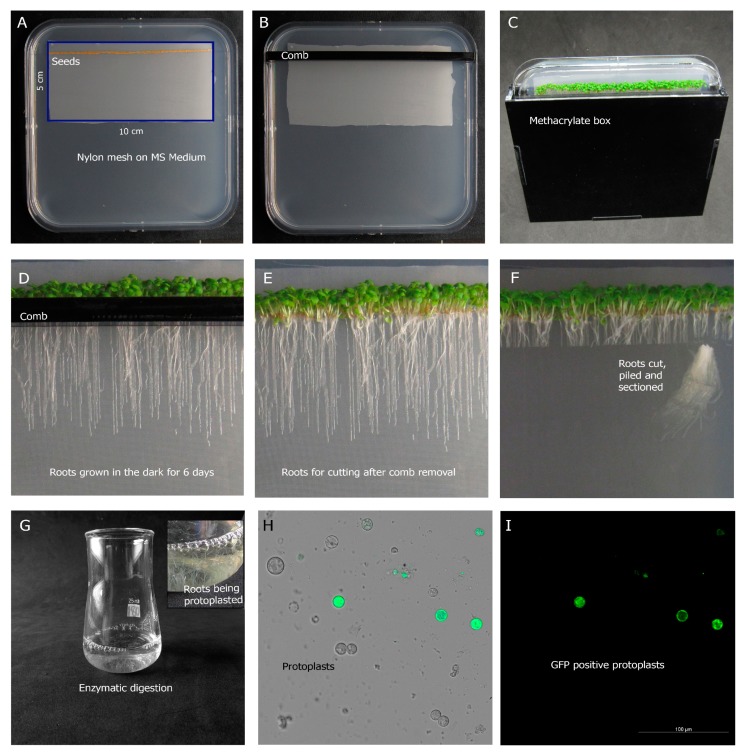
Procedure for seedling growth in the D-Root device, protoplasting, and microscopy analysis before fluorescent activated cell sorting (FACS). (**A**) After sterilization, fluorescent marker line seeds were sown in a row on a sterile Nitex mesh (5 × 10 cm). (**B**) A methacrylate comb was inserted into the agar. (**C**) The Petri dish was inserted into the methacrylate box and grown in standard long-day photoperiodic conditions at 22 °C for 6 days. (**D**–**F**) Removal of the metacrylate box, petri dish lid (**D**), and comb (**E**) prior protoplast processing. (**F**) Roots were cut 1 cm from the hypocotyl, piled, and sectioned into 3 pieces. (**G**) Root sections were collected and incubated with an enzyme mix solution in a small Erlenmeyer flask for 1 hour and 45 minutes. Inset: magnification of the Erlenmeyer flask showing root sections in solution. (**H**,**I**) Protoplasts were examined by fluorescence microscopy. (**H**) Merge of bright field and green fluorescent protein (GFP) settings. (**I**) GFP signal.

Thus, five D-Root plates were harvested and processed to obtain protoplasts (Figure 1A–G, Protocol Section A,B) using solution 1. An aliquot of these protoplasts was examined in a fluorescent microscope under bright and GFP settings to validate protoplast integrity (Figure 1H,I).

**Figure 2:**
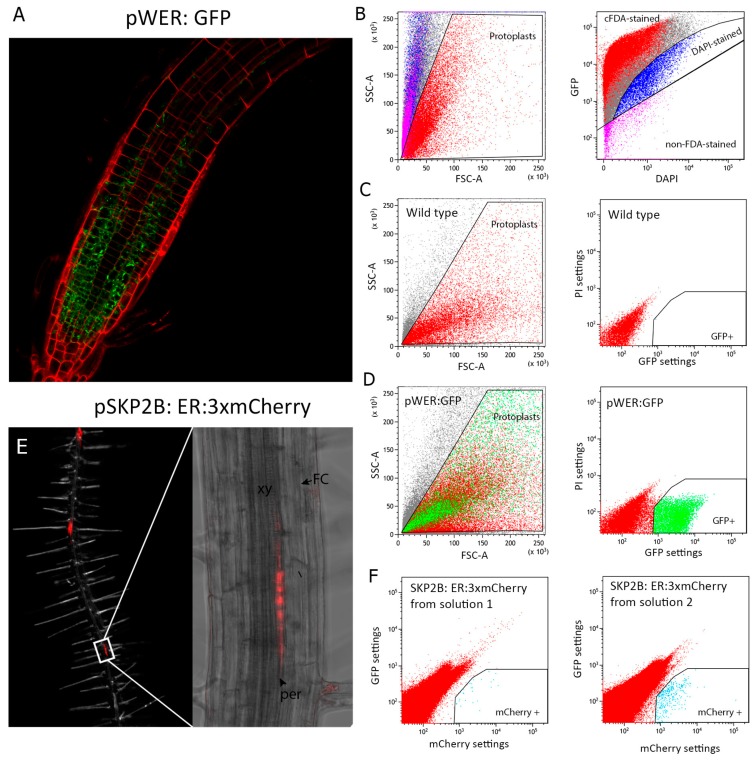
Fluorescence-Activated Cell Sorting (FACS) of root protoplasts. (**A**) Confocal image of plants carrying the fluorescent reporter construct *pWER:ER-GFP* which marks epidermal cells. (**B**) Left panel: Graphic of forward scatter area (FSC-A, x-axis) versus side scatter area (SSC-A, y-axis) for wild type (nonfluorescent control) protoplast events. Right panel: Plot of GFP fluorescence versus DAPI fluorescence. All samples were stained with cFDA and DAPI and analyzed by FACS. The main subpopulations, corresponding to intact viable protoplasts (red, cFDA-stained, and non-DAPI stained), cells whose membranes were not intact (blue, DAPI-stained), and unviable subpopulations (pink, non-FDA-stained protoplasts) were defined. These subpopulations are shown on the left panel by colors. (**C**) Left: Graphic of forward scatter area (FSC-A, x-axis) versus side scatter area (SSC-A, y-axis) for wild type (non-fluorescent control) protoplast events. Right panel: Plot of propidium iodide (PI) fluorescence versus GFP fluorescence. A gate for GFP-positive (GFP+) protoplast collection was defined. (**D**) Left panel: FSC-A/SSC-A plot of *pWER:ER-GFP* line protoplast events. Right: PI signal versus GFP fluorescence plot of *pWER:ER-GFP* protoplast. (**E**) *pSKP2b_0.5Kb_:ER-3xmCherry* line expression in lateral root founder cells; FC: Founder cell, per: pericycle, xy: xylem. (**F**) Green versus mCherry signal plot of *pSKP2B_0.5_:ER-3xmCherry* viable protoplasts during FACS, using solution 1 (Left panel) or solution 2 (Right panel). Light blue dots: mCherry-positive protoplasts.

Next, we processed protoplasts through FACS (Protocol Section C). In order to discriminate between viable protoplasts and debris, we defined a gate within the graph of forward scatter area FSC-A (x-axis) versus side scatter area SSC-A (y-axis) of detected events that we called protoplasts (Figure 2A,B). This area was primarily defined based on cFDA staining, but it also corresponded to cells not stained with DAPI. cFDA staining has been positively associated to membrane integrity whereas DAPI staining reveals defects in membrane integrity [27]. To establish this gate we established the limits of the whole events/population using wild type samples without cFDA staining, which represented a negative non-stained control. Upon cFDA staining, those events still remaining in this area corresponded to “non-FDA staining” events (pink gate), representing dead cells (Figure 2B, right panel). Before staining with DAPI, we also established a limit for cFDA stained cells. Upon double staining with cFDA and DAPI, we determined those cells whose membranes might not be intact (DAPI-stained, in blue). As can be observed in the left panel of Figure 2B, almost all (86.43%) stained events with cFDA and nonstained with DAPI (in red) fell into the gate defined as “protoplasts” and corresponded to viable cells whose membrane would be intact.

In our dark-grown roots conditions, cell death was 6.23% while “DAPI-stained” cells represented 7.34% of the whole population. When light-grown seedlings were used, we found a lower percentage of viable protoplasts (71.46%) (Figure S1), suggesting that the use of the D-Root device, which prevents light stress in roots, might increase the percentage of viable root protoplasts.

To separate fluorescently marked protoplast from autofluorescence, we plotted green (530 nm) signal versus (695 nm) red signal for the GFP-marker lines. This plot enables us to distinguish auto-fluorescence, which should be observed across most of the emission spectrum, in contrast with specific signals generated from the GFP that have a defined emission wavelength range. In a negative control (Figure 2C), we observed that protoplasts were plotted following the diagonal, which indicated that autofluorescence can be detected (as expected) both under green and PI settings. This representation also permits the definition of a gate (in green) for collection of GFP-marked protoplasts (Figure 2C,D). Using this gate, we collected protoplasts to obtain a maximum of 10,000 GFP-positive protoplasts. Lines such as J0661 only generated about 2000 positive protoplasts from the five plates processed. In contrast, the yield for the *pWER:ER-GFP* line was much higher, with 28.1% of the total protoplasts being GFP-positive. 

We determined the minimal amount of enzyme concentration needed. After analyzing 400,000 protoplasts of the J0661 line by FACS, we detected 19,790 GFP-positive protoplasts (12.53%) when the proposed concentration of enzyme mixture was used versus only 1062 GFP-positive protoplasts (0.41%) when half the concentration of enzymes was used (Figure S1B,C). In addition, we also analyzed the percentage of GFP-positive protoplasts of the line J0661 in light-grown root conditions and found a similar percentage, although slightly lower, (10.12%) as compared to when roots were grown in the dark using the D-root device (Figure S1B,D).

In our protocol, we determined that for the more accessible cell types (those listed above), pectolyase was not required to obtain at least 2000 positive protoplasts (all results reported above are without pectolyase). As pectolyase is an expensive enzyme, the costs per experiment could be significantly reduced. Thus, in solution 1, pectolyase was not used. Next, we decided to analyze whether we could sort very scarce cell types located in more differentiated inner root tissues using this nonexpensive solution. The pSKP2B_0.5_ promoter has been previously shown to specifically mark founder cells and other initial cell types during lateral root formations, which are specified inside differentiated or mature sections of the root [35]. Thus, we used the pSKP2B_0.5_ promoter fused to the mCherry to generate *pSKP2B_0.5_:ER-3xmCherry* (Figure 2E). When we tested the *pSKP2B_0.5_:ER-3xmCherry* line, we observed a very low number of extracted protoplasts using solution 1. Even when we increased the number of plates processed (up to 20 plates) and applied pipetting at the end of the digestion, a low number of positive protoplasts were obtained (Figure 2F, left panel). Using solution 2, which contained pectolyase Y-23, in combination with pipetting at the end of the digestion, notably increased the number of positive protoplasts (Figure 2F, right panel).

**Figure 3:**
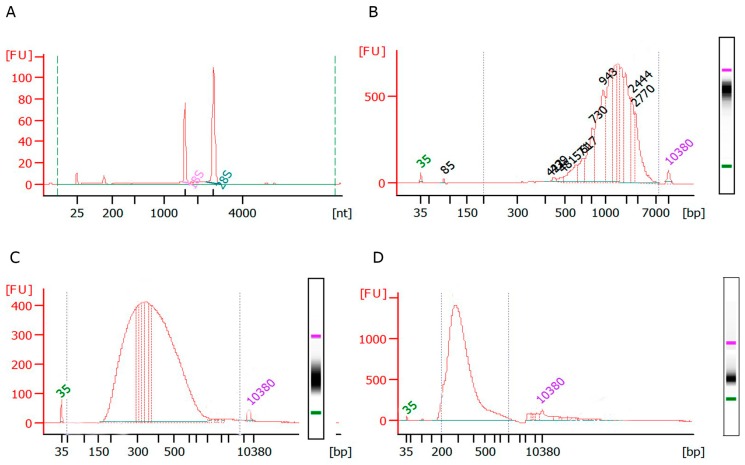
Bioanalyzer analysis of RNA, cDNA, and sequencing libraries generated from GFP- and mCherry-positive protoplasts. (**A**) Electropherogram of an RNA profile extracted from GFP-positive protoplasts (RIN: 9.9, Ratio 28S/18S: 2). (**B**) Profile of cDNA generated from RNA extracted from GFP-positive protoplasts, showing an average size of 1546 bp. Inset: digital electrophoresis. (**C**,**D**) Profile of a sequencing library generated from GFP-positive protoplasts, showing an average size of 400 bp (**C**) or from mCherry-positive protoplasts, showing an average size of 300 bp (**D**). Insets: digital electrophoreses.

This protocol also provides the procedure for RNA extraction (Protocol Section D). The RNA extracted using *pWER:ER-GFP* positive protoplasts fulfilled all RNA criterion parameters (RIN: 9.9, Ratio 28S/18S: 2) when analyzed using a bioanalyzer (Figure 3A). This RNA was used to successfully generate cDNA (Figure 3B), which showed a range of base pairs from 500 to 3000, with 1546 bp being the average size. Next, fragmented cDNA using Covaris was used to generate a library for next-generation sequencing. This fragmented cDNA showed a correct pattern of 200–400 bp average size (Figure 3C). In addition, RNA quality was also assessed using LabChip (RNA Quality Score: 9.5) and TapeStation (RIN: 9) (Figure S2) from 380 mCherry-positive protoplasts of the *pSKP2B0.5Kb:ER-3xmCherry* line. This RNA was processed similarly to successfully obtain a next-generation sequencing library (Figure 3D).

## 3. Protocol: Isolation of Fluorescently Marked Arabidopsis Root Protoplasts from Roots Grown under Darkness Conditions Using D-Root

*A. Plant Growth Using a D-Root Device*

1. Weigh 50 mg of Arabidopsis seeds carrying the desired fluorescent cell type marker. In this case, GFP or mCherry markers were used. *Please note that protoplasting and sorting procedure might have effects on gene expression. It is recommended to perform a comparison between whole roots and processed roots to determine these effects under your experimental settings.*
2. Add 2 mL of 70% bleach solution and mix continuously for 5 min in a laminar flow hood. Pipette off the bleach.
3. Wash the seeds five times with sterile water until clean.
4. Prepare squared plates (12 × 12 cm) filled with 60 mL of an agar-containing medium. This will render similar thickness to all plates and the aperture between the agar and the comb will be about 2 mm, which is enough to permit the growth of roots from the Arabidopsis seeds. You can find additional information of interest in the original publication [17] and in Video S1: Assembling the D-Root system.
5. Place a sterile Nitex mesh (SEFAR Nitex 03-100/44) (10 × 5-mm) into a half Murashige and Skoog basal medium plate. The mesh must be autoclaved before use. We recommend autoclaving the mesh twice, especially for long treatments.
6. Plate the seeds onto the mesh at a density of 3 seeds thickness (90–120 seeds per plate) with a Pasteur pipette.
7. Introduce the petri dish into the D-Root device and draw a line at the top of the D-Root system. Insert a methacrylate comb into the agar in the line you have previously drawn.
8. Close the lid of the plate with micropore tape and store the plates in dark conditions for 3 days at 4 °C to ensure a uniform germination.
9. Put the plates inside the D-Root device at the germination chamber with a growth period of 16 h light (107.45 µmol/m²s)/8 h dark at 22 °C. Note the D-Root device and the methacrylate comb (8 mm thick) have to be sterilized. For this sterilization, we used ozone in an airtight container for 30 min.
10. Grow the roots along the mesh for 6 days.

*B. Protoplasting Procedure*

11. Using a surgical blade, make a first cut that covers all the material and harvest it. Subsequently, and depending on the tissue, make an additional 2 or 3 more cuts to increase the contact between the enzymes and tissues. This additional step increases the protoplast extraction yield and enzyme accessibility to inner tissues, such as pericycle or vascular tissues, that otherwise are hardly reached during the digestion process.
12. Transfer the collected tissue to a small Enlermeyer flask and submerge the pieces into 10 mL protoplasting solution for 1 h and 45 min in an orbital shaker at 100 rpm. Arabidopsis **protoplasting solution (1):** (10 mL): 400 mM mannitol (0.73 g), 20 mM MES hydrate (39 mg), 20 mM KCl (200 μL of 1 M KCl), 1.25% Cellulase R10 (125 mg), and 0.3% Macerozyme R10 (30 mg) in 10 mL water. Adjust pH to 5.7 with 1 M Tris pH 8. Heat 10 min in 55 °C water bath until solution is clear, cool down to room temperature while stirring. Add 10 mM CaCl2 (100 μL of 1 M CaCl2), 0.1% BSA (10 mg), and 1.79 μL of β-mercapoethanol. Arabidopsis **protoplasting solution (2):** This solution only differs from solution 1 in the addition of an extra enzyme, Pectolyase Y-23 0.35%. Both solutions should be made fresh, the same day that the protoplasts are prepared. If the material was previously treated with chemicals or hormones, such as auxins, cytokinins, estradiol, etc., the protoplasting solution is recommended to be supplemented with the same chemicals and hormones. This does not affect protoplast extraction and should ensure effectiveness of the treatment.
13. After 1 h and 45 min of incubation, pipette up and down vigorously using a P1000 micropipette set to 800 μL. This step is critical for extracting scarce and non-accessible root cell populations.
14. Filter the contents of the Enlermeyer flasks through a 70 μm strainer (Fisher scientific # 352350, Whaltham, MA, USA) into a 50 mL Falcon. For meristematic cell types, it is also recommended to additionally filter the protoplast preparation through a 40 μm strainer (Fisher scientific # 352340, Whaltham, MA, USA) into a 50 mL Falcon tube.
15. Centrifuge the Falcon tubes at 500× *g* for 10 min at room temperature.
16. Aspirate and discard the supernatant except 500 μL. Carefully re-suspend protoplast pellet into the remaining supernatant using a P1000 micropipette.
17. Use 10 μL of protoplast preparation to check the protoplast integrity under the microscope and appropriate fluorescence settings (e.g., GFP or mCherry setting) before sorting. It is recommended to use a Neubauer counting chamber to avoid protoplast smashing. If the protocol has been performed successfully, individual isolated protoplasts should be observed.

*C. Fluorescently Marked Protoplast Selection through FACS*

18. Separate the GFP or Cherry protoplasts by passing them through a FACS (for instance FACS-Aria (Becton Dickinson) or other) fitted with a 100 μm nozzle and a 20 psi sheath pressure at a flow rate of 1000–2000 events per second.
19. Depending on the tissue, the protoplasts obtained may have different shape and complexity, which is important when you are defining your population gate for the first time. Create a graph of forward scatter area FSC-A (x-axis) versus side scatter area SSC-A (y-axis) and define your population gate taking into account the following: (1) the area where most the viable protoplasts localize based on the absence of DAPI staining or presence of cFDA, (2) the lowest possible complexity (SSC-A), and (3) where most of the fluorescently-marked protoplasts are located. The ideal gate should fulfill these three criteria while corresponding to a cluster of cells in the graph (Figure 2B–D).
20. Once your population gate is generated, create a new dot plot, linked to the population gate, of GFP settings (530 nm) versus PI settings (695 nm) of fluorescence for GFP-expressing lines. Set up a new gate (GFP+) using a negative control (protoplast population without markers) to establish the boundaries of an area without protoplasts. When the protoplasts from your GFP line are loaded into the FACS system, positive GFP protoplasts will fall into the gate you previously generated. Note that this plot enables you to differentiate false GFP-positive protoplasts from the positive ones, as autofluorescence will emit in both axis. For lines expressing red fluorescent proteins, plot mCherry settings (610 nm) versus GFP settings (530 nm). We decided to use red in the case of GFP-marker lines or GFP in the case of mCherry lines, but you can use other wave lengths such as blue or ultraviolet to check the autofluorescence.
21. Using low binding tubes, collect the sorted protoplasts directly into 300 μL of lysis buffer (Qiagen RLT buffer, Germantown, MD, USA) plus 3 μL of ß-mercaptoethanol. Mix well the tube before freezing on dry ice. Although sorted protoplasts may remain in the −80 °C freezer for months, we recommend extracting the RNA as soon as possible.

*D. RNA Extraction for cDNA Synthesis and Sequencing Library Preparation*

22. To isolate total RNA from sorted cells, gently flick the tubes to mix the cells. Note: We performed a successful RNA extraction using 100 positive protoplasts.
23. Add 300 μL of 70% cold ethanol to the homogenized lysate. Mix well by pipetting.
24. Apply 600 μL of the sample including any precipitate to a pink RNEasy column (Quiagen, Germantown, MD, USA) sitting in a 2 mL collection tube. Spin at 1000× *g* for 1 min, then 10,000× *g* for 30 s. Reapply flow-through and spin at 10,000× *g* for 30 s. Reuse collection tube in the next step. Note that other extraction methods such as miRVana (Ambion) or quick-RNA plant Kit (Zymo Research) can be used instead.
25. Pipette 700 μL of buffer RW1 (component of the Quiagen kit ID 74104, Germantown, MD, USA) onto the RNEasy column and centrifuge for 30 s at 10,000× *g*. Discard flow-through.
26. Pipette 500 μL buffer RPE (component of the Quiagen kit ID 74104, Germantown, MD, USA) onto the RNEasy column, and centrifuge for 30 s at 10,000× *g*. Discard flow-through.
27. Pipette 500 μL buffer 80% ethanol. Spin at 10,000× *g* for 2 min to dry the RNEasy membrane. Place column in fresh tube.
28. Centrifuge for 5 min at maximum speed. Put the centrifuge at 18 °C.
29. Place the RNEasy spin column in a new 1.5 mL collection tube and discard the collection tube with the filtrate. Spin at 17,000× *g* for 1 min until you see a drop in the bottom of the tube.
30. Transfer the RNEasy column onto a label (on the top and lateral sides) in a 1.5 mL collection tube and pipette 20 μL of RNAse-free water directly on the RNEasy membrane. Centrifuge for 1 min at 1000× *g* plus, 1 min 17,000× *g* to elute. Repeat this step 1 min 17,000× *g* with the same eluted RNA to increase yield.
31. Determine RNA integrity using the Agilent 6000 RNA Nanokit (Agilent, Santa Clara CA, USA) on an Agilent 2100 Bioanalyzer (Agilent, Santa Clara CA, USA) and Qubit™ RNA Assay Kits (Fisher scientific, Whaltham, MA, USA) on Qubit^®^ 2.0 Fluorometer (Fisher scientific, Whaltham, MA, USA) according to the manufacturer’s protocol.
32. cDNA synthesis and library preparation can then be performed with available kits such as SMART-Seq v4 Ultra Low Input RNA kit (Takara, Mountain View, CA, USA) and The SMARTer^®^ ThruPLEX^®^ DNA-Seq Kit (Takara) or others.

## 4. Discussion

The use of protoplasting combined with FACS technology has enormously contributed to the increase of our knowledge of plant regulatory mechanisms, highlighting those specific to a cellular tissue under different environmental conditions [7,18,19,20,21]. Different fluorescent proteins (GFP, mCherry, CFP, YFP, etc.), as well as their combinations, can be used to distinguish specific cellular populations depending on the characteristics of the FACS. Based on our results, we have combined the use of different markers (cFDA for cell viability and DAPI for loss of cell membrane integrity) to generate a suitable gate for protoplasting. 

Our results indicate that growing the seedlings in dark-grown root conditions does not have a negative influence compared to those grown in light-grown root conditions, on the contrary, dark-grown conditions could be beneficial. Moreover, the percentage of positive protoplasts from dark-grown roots is slightly higher than in light-grown root. Taking all our observations into account, the use of the D-Root system represents an improvement for the analyses of root cell types, since protoplasts can be efficiently obtained and roots are grown in conditions more similar to nature. Thus, using the D-Root device prevents the influence of root illumination in the process under study, which in many cases could have a strong and negative impact [9,36]. Therefore, FACS in combination with the D-Root device is a good solution to disentangle the molecular mechanisms regulating cell types for specific development and responses to adverse conditions.

For cell sorting experiments, the treatment duration and concentration of enzymes are critical factors that should be standardized. The cell wall and the middle lamella contain pectin and cellulase. As the root grows, tissues differentiate through thickening of the cell walls and new physical structures, such as the Casparian strip and suberization, are developed [34]. Casparian strips are lignin impregnations located on the middle lamella between neighbor cells in the endodermis, a layer that surrounds the pericycle and central vascular tissues. In order to extract viable protoplasts from the stele in more differentiated root sections, a very efficient digestion of the cell wall and middle lamella seems to be necessary, as lignin is not easily digested. Digestion of the cell wall and middle lamella can be achieved by using cellulases and pectinases. The next inner layer surrounded by the endodermis is the pericycle, which is the tissue that originates new lateral roots through the specification of founder cells [37,38]. In differentiated and mature zones of the root, pericycle cells are sclerified. In addition, EXPANSIN A1 has been reported to function in pericycle cell wall modification during lateral root initiation, suggesting that the founder cell walls may have special characteristics [33]. Based on these characteristics and because they are surrounded by the endodermis, extracting protoplasts from founder cells might then be more challenging than doing so from other tissues.

In our protocol, we first focused on minimizing the number of enzymes in our digestion mix to optimize costs, depending on the root tissue of interest. Our results demonstrated that cellulase and macerozyme, which are a mix of enzymes from *Rhizopus* sp., are required and sufficient for efficient extraction of positive protoplasts from accessible or abundant cell types. Macerozymes also have multiple actions as pectinase, hemicellulase, and cellulase. Both cellulases and macerozymes are the enzymes we proposed to use in solution 1 to reduce cost while maintaining yield in the more accessible cell types. In contrast, for the innermost tissues of the root-differentiated zone, we propose adding pectolyase Y-23, which is a mix of pectolyase enzymes from *Aspergillus japonicus*. Pectolyase Y-23 has endopolygalacturonase and endopectin lyase activities that increased the efficiency of protoplasting, and thus allowed for the extraction of scarce and/or non-accessible cell types. 

## Supplementary Materials

The following are available online at https://www.mdpi.com/2223-7747/9/4/499/s1, Figure S1: Viability assessment and FACS experiments in dark and light conditions, as well as different enzyme concentrations (A) Graphic representation of the percentage of protoplast viability cFDA (live protoplasts), DAPI (show the loss of membrane integrity) staining and dead cells corresponding to no-stained root cells from seedlings grown with the D-Root device (dark) or without the D-Root (light). Propidium iodine settings/GFP settings plots of J0661 under different conditions: (B) Dark conditions, (C) Dark conditions with half enzymes and (D) Light conditions. Figure S2: Analysis of RNA and cDNA from mCherry sorted positive protoplasts. (A) Electropherogram of RNA profile from 380 mCherry positive protoplasts using LabChip (RNA Quality Score 9) and (B) TapeStation technology (RIN: 9). (C) Bio-analyzer profile of cDNA showing an average size of 1700 bp. Inset: digital electrophoresis.

## References

[1] Castrillo G., Teixeira P.J., Paredes S.H., Law T.F., de Lorenzo L., Feltcher M.E., Finkel O.M., Breakfield N.W., Mieczkowski P., Jones C.D. (2017). Root microbiota drive direct integration of phosphate stress and immunity. Nature.

[2] Koevoets I.T., Venema J.H., Elzenga J.T., Testerink C. (2016). Roots Withstanding their Environment: Exploiting Root System Architecture Responses to Abiotic Stress to Improve Crop Tolerance. Front. Plant Sci..

[3] Aloni R., Aloni E., Langhans M., Ullrich C.I. (2006). Role of cytokinin and auxin in shaping root architecture: Regulating vascular differentiation, lateral root initiation, root apical dominance and root gravitropism. Ann. Bot..

[4] Bhosale R., Giri J., Pandey B.K., Giehl R.F.H., Hartmann A., Traini R., Truskina J., Leftley N., Hanlon M., Swarup K. (2018). A mechanistic framework for auxin dependent Arabidopsis root hair elongation to low external phosphate. Nat. Commun..

[5] Liu J., Moore S., Chen C., Lindsey K. (2017). Crosstalk Complexities between Auxin, Cytokinin, and Ethylene in Arabidopsis Root Development: From Experiments to Systems Modeling, and Back Again. Mol. Plant.

[6] Himanen K., Vuylsteke M., Vanneste S., Vercruysse S., Boucheron E., Alard P., Chriqui D., Van Montagu M., Inze D., Beeckman T. (2004). Transcript profiling of early lateral root initiation. Proc. Natl. Acad. Sci. USA.

[7] Brady S.M., Orlando D.A., Lee J.Y., Wang J.Y., Koch J., Dinneny J.R., Mace D., Ohler U., Benfey P.N. (2007). A high-resolution root spatiotemporal map reveals dominant expression patterns. Science.

[8] Iyer-Pascuzzi A., Simpson J., Herrera-Estrella L., Benfey P.N. (2009). Functional genomics of root growth and development in Arabidopsis. Curr. Opin. Plant. Biol..

[9] Silva-Navas J., Moreno-Risueno M.A., Manzano C., Tellez-Robledo B., Navarro-Neila S., Carrasco V., Pollmann S., Gallego F.J., Del Pozo J.C. (2016). Flavonols Mediate Root Phototropism and Growth through Regulation of Proliferation-to-Differentiation Transition. Plant Cell.

[10] Tellez-Robledo B., Manzano C., Saez A., Navarro-Neila S., Silva-Navas J., de Lorenzo L., Gonzalez-Garcia M.P., Toribio R., Hunt A.G., Baigorri R. (2019). The polyadenylation factor FIP1 is important for plant development and root responses to abiotic stresses. Plant J. Cell Mol. Biol..

[11] Swarup R., Parry G., Graham N., Allen T., Bennett M. (2002). Auxin cross-talk: Integration of signalling pathways to control plant development. Plant Mol. Biol..

[12] Halliday K.J., Martinez-Garcia J.F., Josse E.M. (2009). Integration of light and auxin signaling. Cold Spring Harb. Perspect. Biol..

[13] Dyachok J., Zhu L., Liao F., He J., Huq E., Blancaflor E.B. (2011). SCAR mediates light-induced root elongation in Arabidopsis through photoreceptors and proteasomes. Plant Cell.

[14] Laxmi A., Pan J., Morsy M., Chen R. (2008). Light plays an essential role in intracellular distribution of auxin efflux carrier PIN2 in Arabidopsis thaliana. PLoS ONE.

[15] Sassi M., Lu Y., Zhang Y., Wang J., Dhonukshe P., Blilou I., Dai M., Li J., Gong X., Jaillais Y. (2012). COP1 mediates the coordination of root and shoot growth by light through modulation of PIN1- and PIN2-dependent auxin transport in Arabidopsis. Development.

[16] Xu W., Ding G., Yokawa K., Baluska F., Li Q.F., Liu Y., Shi W., Liang J., Zhang J. (2013). An improved agar-plate method for studying root growth and response of Arabidopsis thaliana. Sci. Rep..

[17] Silva-Navas J., Moreno-Risueno M.A., Manzano C., Pallero-Baena M., Navarro-Neila S., Téllez-Robledo B., Garcia-Mina J.M., Baigorri R., Javier Gallego F., del Pozo J.C. (2015). D-Root: A system to cultivate plants with the root in darkness or under different light conditions. Plant J..

[18] Birnbaum K., Shasha D.E., Wang J.Y., Jung J.W., Lambert G.M., Galbraith D.W., Benfey P.N. (2003). A gene expression map of the Arabidopsis root. Science.

[19] Nawy T., Lee J.Y., Colinas J., Wang J.Y., Thongrod S.C., Malamy J.E., Birnbaum K., Benfey P.N. (2005). Transcriptional profile of the Arabidopsis root quiescent center. Plant Cell.

[20] Lee J.Y., Colinas J., Wang J.Y., Mace D., Ohler U., Benfey P.N. (2006). Transcriptional and posttranscriptional regulation of transcription factor expression in Arabidopsis roots. Proc. Natl. Acad. Sci. USA.

[21] Levesque M.P., Vernoux T., Busch W., Cui H., Wang J.Y., Blilou I., Hassan H., Nakajima K., Matsumoto N., Lohmann J.U. (2006). Whole-genome analysis of the SHORT-ROOT developmental pathway in Arabidopsis. PLoS Biol..

[22] Clark N.M., Fisher A.P., Sozzani R. (2018). Identifying Differentially Expressed Genes Using Fluorescence-Activated Cell Sorting (FACS) and RNA Sequencing from Low Input Samples. Methods Mol. Biol. (Clifton N.J.).

[23] Redenbaugh K., Ruzin S., Bartholomew J., Bassham J.A. (1982). Characterization and separation of plant protoplasts via flow cytometry and cell sorting. Z. Für Pflanzenphysiol..

[24] Hammatt N., Lister A., Blackhall N., Gartland J., Ghose T., Gilmour D., Power J., Davey M., Cocking E. (1990). Selection of plant heterokaryons from diverse origins by flow cytometry. Protoplasma.

[25] Galbraith D.W., Lambert G.M., Grebenok R.J., Sheen J. (1995). Flow cytometric analysis of transgene expression in higher plants: Green-fluorescent protein. Methods Cell Biol..

[26] Galbraith D.W. (2014). Flow cytometry and sorting in Arabidopsis. Methods Mol. Biol. (Clifton N.J.).

[27] Amor K.B., Breeuwer P., Verbaarschot P., Rombouts F.M., Akkermans A.D., De Vos W.M., Abee T. (2002). Multiparametric flow cytometry and cell sorting for the assessment of viable, injured, and dead Bifidobacterium cells during bile salt stress. Appl. Environ. Microbiol..

[28] Guzzo F., Cantamessa K., Portaluppi P., Levi M. (2002). Flow cytometry and sorting of protoplasts from carrot cell cultures reveal two cell subpopulations with different morphogenetic potential. Plant Cell Rep..

[29] Vilarrasa-Blasi J., Gonzalez-Garcia M.P., Frigola D., Fabregas N., Alexiou K.G., Lopez-Bigas N., Rivas S., Jauneau A., Lohmann J.U., Benfey P.N. (2014). Regulation of plant stem cell quiescence by a brassinosteroid signaling module. Dev. Cell.

[30] Dinneny J.R., Long T.A., Wang J.Y., Jung J.W., Mace D., Pointer S., Barron C., Brady S.M., Schiefelbein J., Benfey P.N. (2008). Cell identity mediates the response of Arabidopsis roots to abiotic stress. Science.

[31] Bargmann B.O., Birnbaum K.D. (2010). Fluorescence activated cell sorting of plant protoplasts. J. Vis. Exp. Jove.

[32] Laplaze L., Parizot B., Baker A., Ricaud L., Martiniere A., Auguy F., Franche C., Nussaume L., Bogusz D., Haseloff J. (2005). GAL4-GFP enhancer trap lines for genetic manipulation of lateral root development in Arabidopsis thaliana. J. Exp. Bot..

[33] Ramakrishna P., Ruiz Duarte P., Rance G.A., Schubert M., Vordermaier V., Vu L.D., Murphy E., Vilches Barro A., Swarup K., Moirangthem K. (2019). EXPANSIN A1-mediated radial swelling of pericycle cells positions anticlinal cell divisions during lateral root initiation. Proc. Natl. Acad. Sci. USA.

[34] Naseer S., Lee Y., Lapierre C., Franke R., Nawrath C., Geldner N. (2012). Casparian strip diffusion barrier in Arabidopsis is made of a lignin polymer without suberin. Proc. Natl. Acad. Sci. USA.

[35] Manzano C., Ramirez-Parra E., Casimiro I., Otero S., Desvoyes B., De Rybel B., Beeckman T., Casero P., Gutierrez C., Del Pozo J.C. (2012). Auxin and epigenetic regulation of SKP2B, an F-box that represses lateral root formation. Plant Physiol..

[36] Silva-Navas J., Conesa C.M., Saez A., Navarro-Neila S., Garcia-Mina J.M., Zamarreno A.M., Baigorri R., Swarup R., Del Pozo J.C. (2019). Role of cis-zeatin in root responses to phosphate starvation. New Phytol.

[37] Perez-Garcia P., Moreno-Risueno M.A. (2018). Stem cells and plant regeneration. Dev. Biol..

[38] De Smet I., Vanneste S., Inze D., Beeckman T. (2006). Lateral root initiation or the birth of a new meristem. Plant Mol. Biol..

