# Supplementary material for: Fluorescence-Activated Cell Sorting Using the D-Root Device and Optimization for Scarce and/or Non-Accessible Root Cell Populations
Source: Plants (Basel). 2020 Apr 14;9(4):499. doi: 10.3390/plants9040499 (PMC7238278; doi:10.3390/plants9040499)
Supplement: Supplementary file 1 [file plants-09-00499-s001.pdf]

Supplementary Marerials

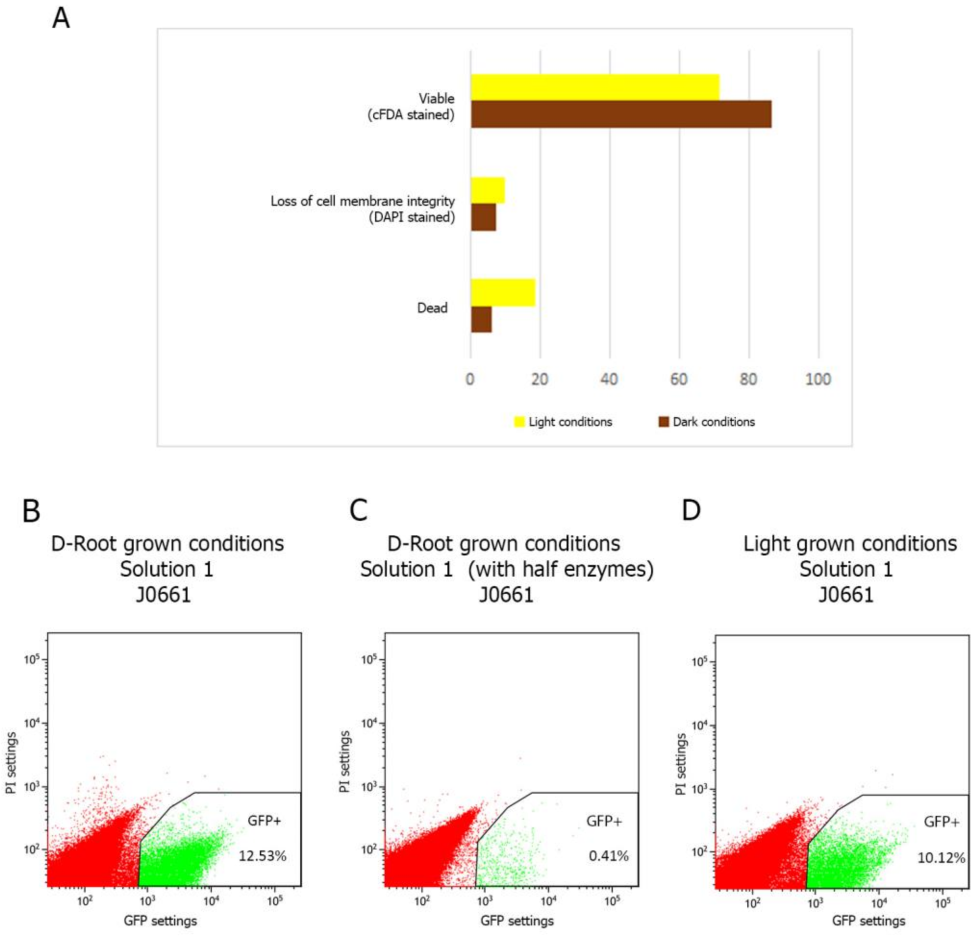

Figure S1. Viability assessment and FACS experiments in dark and light conditions, as well as different enzyme concentrations (**A**) Graphic representation of the percentage of protoplast viability cFDA (live protoplasts), DAPI (show the loss of membrane integrity) staining and dead cells corresponding to no-stained root cells from seedlings grown with the D-Root device (dark) or without the D-Root (light). Propidium iodine settings/GFP settings plots of *J0661* under different conditions: (**B**) Dark conditions, (**C**) Dark conditions with half enzymes and (**D**) Light conditions.

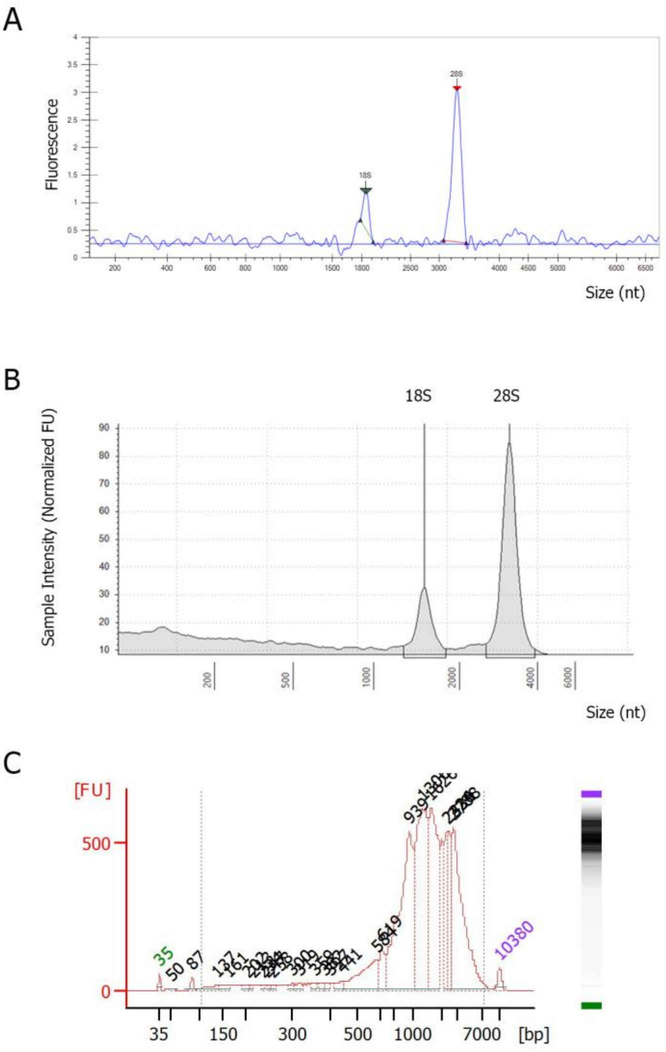

Figure S2. Analysis of RNA and cDNA from mCherry sorted positive protoplasts. (**A**) Electropherogram of RNA profile from 380 mCherry positive protoplasts using LabChip (RNA Quality Score 9) and (**B**) TapeStation technology (RIN: 9). (**C**) Bio-analyzer profile of cDNA showing an average size of 1700 bp. Inset: digital electrophoresis.
